# Supplementary material for: A general strategy for generating expert-guided, simplified views of ontologies
Source: bioRxiv. 2025 Aug 29:2024.12.13.628309. Originally published 2024 Dec 17. Preprint. [Version 2] doi: 10.1101/2024.12.13.628309 (PMC11702530; doi:10.1101/2024.12.13.628309)
Supplement: Supplement 1 [file NIHPP2024.12.13.628309v2-supplement-1.pdf]

bioRxiv preprint doi: https://doi.org/10.1101/2024.12.13.628309; this version posted August 29, 2025. The copyright holder for this preprint (which was not certified by peer review) is the author/funder, who has granted bioRxiv a license to display the preprint in perpetuity. It is made available under aCC-BY 4.0 International license.

Supplementary Figure

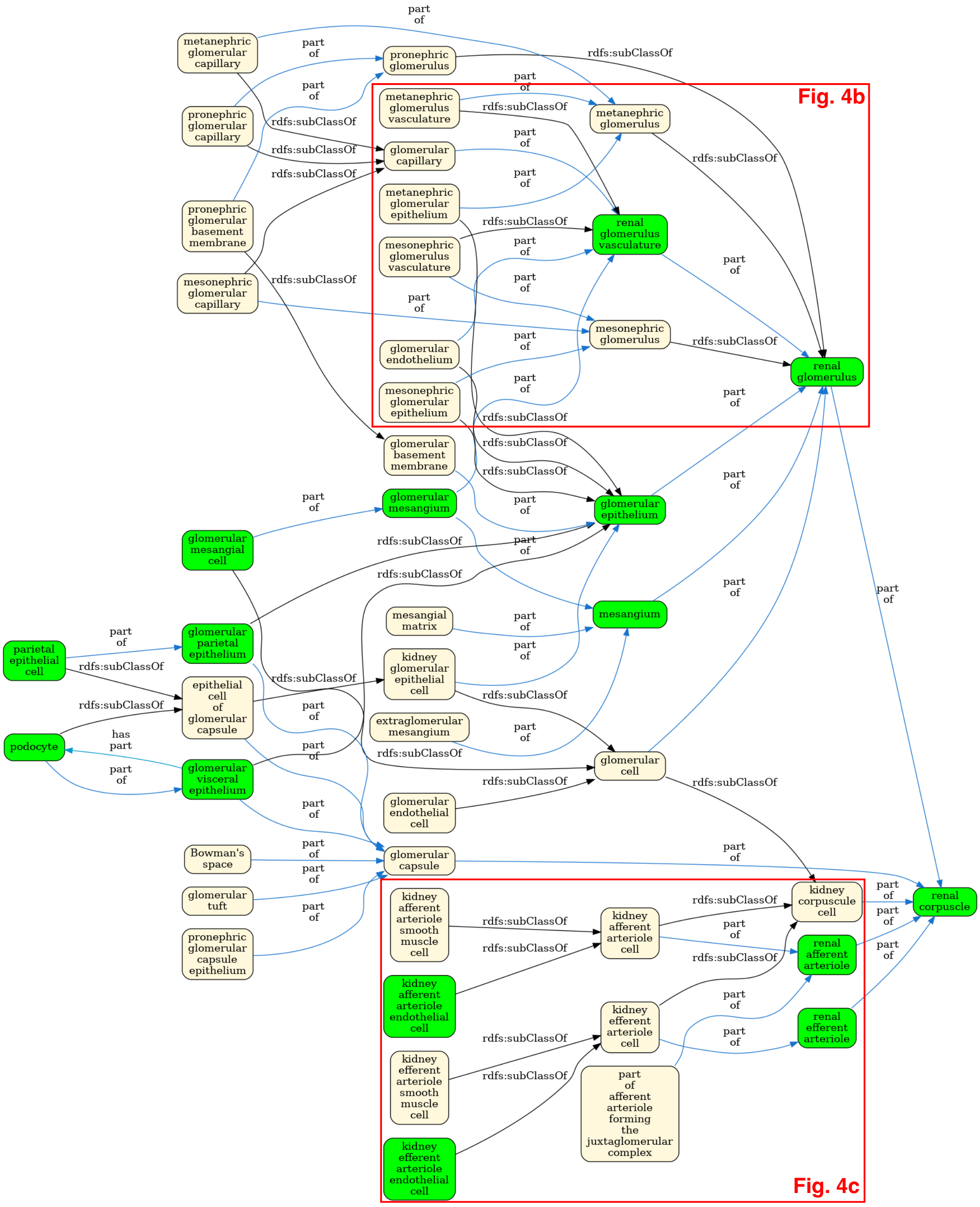

Fig. S1 Uberon/CL ontology graph for the renal corpuscle with terms referenced in the HRA kidney ASCT+B table in green, including the renal corpuscle cell types illustrated in Fig. 4a. This illustrates the complexity of the Uberon graph compared to the needs of the HRA. Enlarged views of selected regions of the graph are provided in Fig. 4b and Fig. 4c.
