# Supplementary material for: A general strategy for generating expert-guided, simplified views of ontologies
Source: bioRxiv. 2024 Dec 17:2024.12.13.628309. Preprint. [Version 1] doi: 10.1101/2024.12.13.628309 (PMC11702530; doi:10.1101/2024.12.13.628309)
Supplement: Supplementary file 1 [file NIHPP2024.12.13.628309v1-supplement-1.pdf]

bioRxiv preprint doi: https://doi.org/10.1101/2024.12.13.628309; this version posted December 18, 2024. The copyright holder for this preprint (which was not certified by peer review) is the author/funder, who has granted bioRxiv a license to display the preprint in perpetuity. It is made available under aCC-BY 4.0 International license.

Supplementary figure

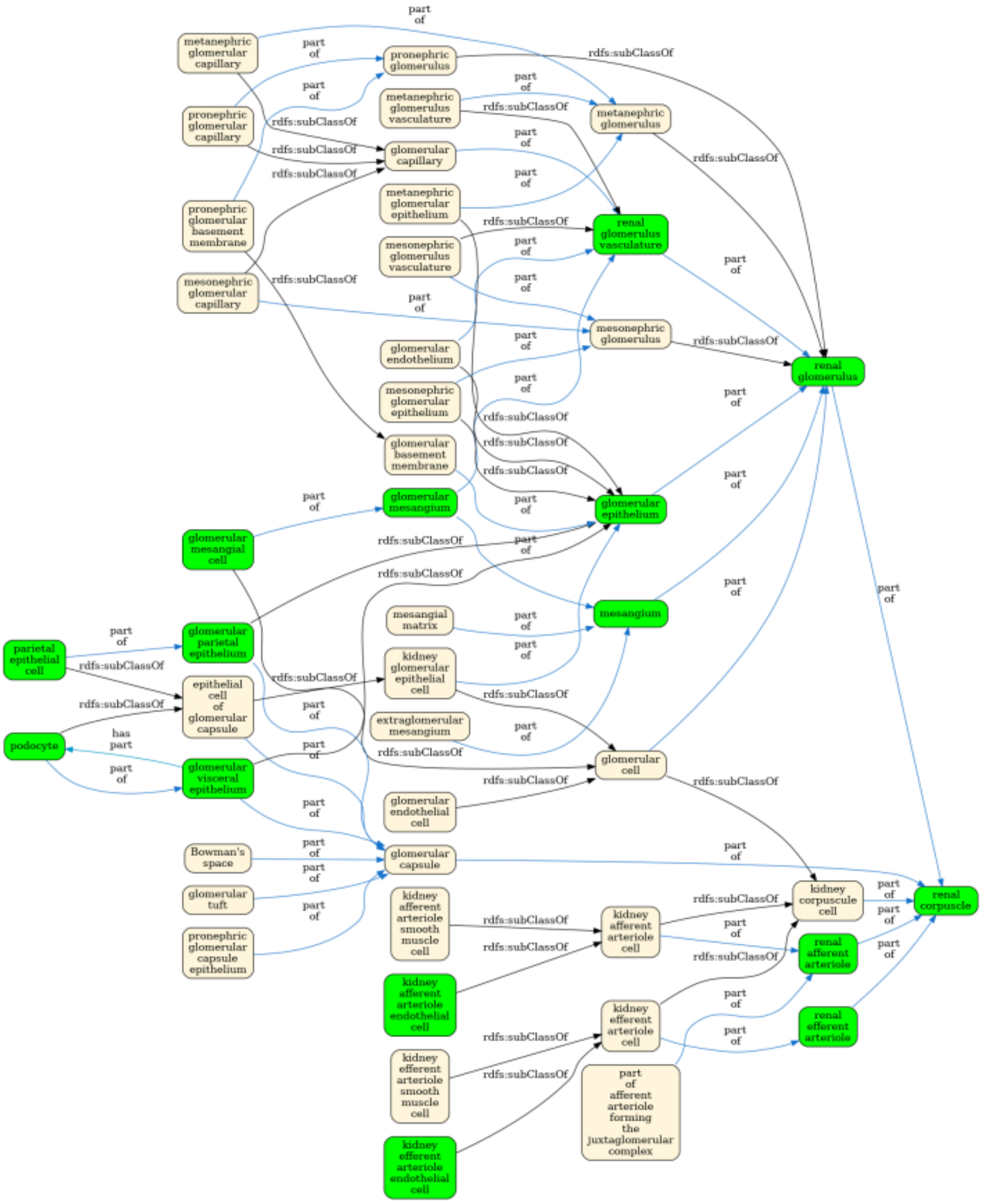

Fig. S1 A detailed view of Fig 2B depicting a subset of all Uberon and CL terms that are part of the renal corpuscle.
